# IDLaS-DE – A Web-Based Platform for Running Customized Studies on Individual Differences in German Language Skills

**DOI:** 10.5334/joc.468

**Published:** 2025-11-10

**Authors:** Sandra Bethke, Janay Monen, Thijs Rinsma, Paul Trilsbeek, Antje S. Meyer, Florian Hintz

**Affiliations:** 1 Max Planck Institute for Psycholinguistics, Nijmegen, The Netherlands; 2 Radboud University, Nijmegen, The Netherlands; 3 Research Center Deutscher Sprachatlas, Marburg University, Germany; 4 Center for Mind, Brain and Behavior, Marburg University & Justus Liebig University, Germany

**Keywords:** individual differences, language skills, online testing, test battery

## Abstract

Individuals vary substantially in their language skills. The *Individual Differences in Language Skills Test Battery* (IDLaS) is a tool to assess variability in (1) linguistic experience, (2) general cognitive skills implicated in language, including nonverbal processing speed, working memory, and nonverbal reasoning, and (3) linguistic processing skills, including word- and sentence-level production and comprehension. The test battery was initially developed for Dutch language users. Building on this work, we recently developed a German version (IDLaS-DE). IDLaS-DE consists of 30 behavioral tests that have been validated in a large group of German speakers, aged between 18 and 30 years. In addition, we have developed a web platform that researchers interested in assessing language and general cognitive skills can use for their research purposes. Here, we provide a guide for creating and running customized studies online via this platform. The IDLaS-DE web platform and all its services are free of charge and accessible at https://www.mpi.nl/idlas-de.

## Introduction

Individuals differ greatly in their ability to use language. However, psycholinguistics has only recently begun to systematically examine this variability in healthy adult speakers. To that end, researchers have started to incorporate tests capturing individual differences into their experimental designs or assembled whole batteries of individual differences tests. However, investigating individual differences remains challenging for several reasons. Many factors may influence participants’ performance on a behavioral test. A given test will, thus, never provide a pure estimate of one underlying skill. For example, a timed word identification task may measure participants’ knowledge of words, but also their response speed. This problem has been widely recognized in other fields and is referred to as task impurity problem. Task impurity often results in the conflation of skills, since complex skills tend to be correlated when the tests assessing these skills share task components and therefore underlying cognitive processes, such as making a choice or giving a timed response (Hintz et al., 2025; Miyake et al., 2000). This can be seen, for instance, in the correlation between working memory capacity and measures of IQ (Conway et al., 2012), or in the correlation between speakers’ vocabulary size and their grammatical knowledge (Dąbrowska, 2018; Street & Dąbrowska, 2010).

To address task impurity, the skill of interest needs to be tested using multiple tests. Moreover, skills likely to be correlated with the skill of interest need to be assessed as well, such that correlations between skills can be identified and taken into account in statistical analyses and theory building. To measure skills systematically, researchers have assembled batteries of tests assessing multiple skills with multiple tests per skill, respectively. Such test batteries are used to study patterns of individual variability. However, to detect and draw meaningful conclusions about such individual variation, a large number of participants needs to be assessed (Brysbaert, 2024; Schönbrodt & Perugini, 2013).

In practice, it is rarely possible for researchers to assess a skill of interest in conjunction with all potentially overlapping traits. A more feasible research strategy is to assess those skills that are most likely to overlap and are of key theoretical interest. This strategy has been used in a number of studies on language skills. There is consensus that language processing involves not only linguistic skills, such as vocabulary size or grammatical knowledge, but also general cognitive abilities, such as nonverbal reasoning or working memory. Thus, some researchers have incorporated both, linguistic and non-linguistic skills, in their studies. Dąbrowska (2018), for instance, investigated various aspects of linguistic knowledge, using multiple tests that assess vocabulary size, grammatical comprehension, print exposure and collocational knowledge, and related these linguistic skills to measures of nonverbal intelligence. Kuperman et al. (2018) utilized tests assessing general cognitive abilities and executive functioning skills, and related them to measures of reading comprehension, again using multiple tests for each domain. In a multimodal study, Gillespie et al. (2014) investigated how differences in working memory capacity and lexical retrieval skills related to participants’ performance in a gesture elicitation task.

Most of the studies assessing individual differences in language skills have been conducted in English. Our goal is to facilitate such comprehensive studies, in particular for German speakers. So far, there have only been a few studies conducted in German. For example, Amunts and colleagues (2021) tested 230 German speakers on a battery of 14 tests of executive functions alongside three verbal fluency tasks. Similarly, Camilleri and colleagues (2024) tested 148 German speakers on the same set of executive functioning tests together with four speech production tasks (including verbal fluency). The latter data set is available for research purposes upon request. The current work is complementary to these studies. We make available a large battery of tests to the research community to assess key language skills and general cognitive skills involved in language processing.

The *Individual Differences in Language Skills Test Battery* for German (IDLaS-DE) contains 30 behavioral tests assessing individual differences in language-related skills. It was designed for German speaking adults between 18 and 30 years. This test battery was translated and adapted from the Dutch prototype, the *Individual Differences in Language Skills Test Battery* for Dutch speakers (IDLaS-NL), which was validated in the same age group (Hintz et al., 2025). Such cross-linguistic test adaptations are not only an efficient tool for test development (Bermúdez-Margaretto & Brysbaert, 2025), but also contribute to standardizing test procedures in individual differences research across labs and languages.

The behavioral tests in both, IDLaS-DE and IDLaS-NL, cover eight psychological constructs involved in language processing: (1) linguistic experience, (2) processing speed, (3) working memory, (4) nonverbal reasoning, (5) word comprehension, (6) word production, (7) sentence comprehension and (8) sentence production. An overview of the 30 tests in IDLaS-DE is displayed in Table 1. Twenty-two of these have been directly adapted from IDLaS-NL, which contains a total of 35 tests. Another three tests that have previously been developed for German speakers by other labs were included, as they were equivalents to the tests used in IDLaS-NL (German Author Recognition Test, Hagener Matrizentest, Big Five Inventory 10). References are listed in the source column of Table 1. One sentence comprehension and one word comprehension test of the Dutch battery (the monitoring in noise tasks) were omitted in IDLaS-DE, as they showed weak loadings on the sentence comprehension factor in a confirmatory factor analysis (Hintz et al., 2025). The IDLaS-NL web platform offers six additional tests that were developed in other labs. As these are not considered part of the test battery, they were not translated into German. Four tests (labelled as “newly developed” in Table 1) were designed for IDLaS-DE and do not occur in IDLaS-NL. These tests were developed to substitute the tests that were omitted from IDLaS-NL. For instance, two sentence generation tests from IDLaS-NL were not included in the German version, since the image stimuli were not usable for the German version. However, some of the items of both tests were combined into a new sentence generation test for IDLaS-DE.

**Table 1:** Descriptions of the 30 tests included in IDLaS-DE.


	DOMAIN	TEST	TASK DESCRIPTION	PERFORMANCE INDICATOR	DURATION	SOURCE

1	Linguistic experience	German Auditory & Image Vocabulary Test	Participants hear a spoken word and select the image associated with its meaning out of four alternatives.	Accuracy	15 min	Bethke et al. (2025)

2		Untimed antonym production (productive vocabulary)	Participants hear a spoken word and are instructed to produce its antonym.	Accuracy	5 min	adapted from IDLaS-NL

3		Idiom recognition test	Participants select the correct meaning for an idiomatic expression among four alternatives.	Accuracy	3 min	adapted from IDLaS-NL

4		Spelling test	Participants identify incorrectly spelled words in a list of 60 words (half of which are spelled incorrectly).	Accuracy	5 min	adapted from IDLaS-NL

5		German Author Recognition Test	Participants identify real authors (fiction writers) in a list of 125 names (75 are writers).	Accuracy	5 min	Groliget al. (2020)

6		Book Language Grammar test	Participants carry out grammaticality judgements on spoken sentences featuring morpho-syntactic constructions known to be difficult for adult speakers of German (e.g., “wie” vs. “als”, “je…desto”).	Accuracy	10 min	adapted from IDLaS-NL

7	Processing speed	Auditory simple reaction time	Participants respond as quickly as possible to the presentation of a beep by pressing the space bar.	Reaction time	3 min	adapted from IDLaS-NL

8		Auditory choice reaction time	Participants respond as quickly as possible to the presentation of one of two beeps (high or low) by pressing one of two buttons.	Reaction time	5 min	adapted from IDLaS-NL

9		Letter comparison	Participants indicate as quickly as possible whether two letter strings are identical by pressing one of two buttons.	Reaction time	5 min	adapted from IDLaS-NL

10		Visual simple reaction time	Participants respond as quickly as possible to the presentation of a geometrical shape by pressing the space bar.	Reaction time	3 min	adapted from IDLaS-NL

11		Visual choice reaction time	Participants respond as quickly as possible to the presentation of one of two geometrical shapes by pressing one of two buttons.	Reaction time	5 min	adapted from IDLaS-NL

12	Working memory	Digit span	Participants are instructed to recall sequences of spoken digits in the order they appeared (forward version) or in the reversed order (backward version).	Accuracy	7 min	adapted from IDLaS-NL

13		Corsi block clicking	Participants are instructed to recall sequences of identical spatially separated blocks by clicking on them in the order they appeared (forward version) and in the reversed order (backward version).	Accuracy	7 min	adapted from IDLaS-NL

14	Nonverbal reasoning	Hagener Matrizentest	Participants select a shape among six alternatives that completes a matrix of geometric patterns.	Accuracy	20 min	Heydasch & Schnaedter (2014)

15	Word production	Picture naming	Participants name pictures whose names vary in word frequency as quickly as possible.	Reaction time	7 min	adapted from IDLaS-NL

16		Rapid automatized naming	Participants are familiarized with four sets of five objects whose names vary orthogonally in frequency and neighborhood density. Each object set is arranged in an array consisting of five rows of six objects. Participants have to name all objects in the array as quickly as possible.	Reaction time	7 min	adapted from IDLaS-NL

17		Verbal fluency	Within one minute, participants name as many words as possible belonging to pre-specified categories (semantic version) or starting with letters provided ahead of time (phonological version).	Accuracy	5 min	adapted from IDLaS-NL

18		Timed antonym production	Participants hear a spoken word and have to produce its antonym as quickly as possible within four seconds.	Reaction time	5 min	newly developed

19		Maximal speech rate	Participants are instructed to name the months of the year as quickly as possible.	Reaction time	3 min	adapted from IDLaS-NL

20	Sentence production	Phrase generation	Participants are familiarized with a set of 16 common objects. Either single objects or combinations of these objects in different colors are then presented to elicit noun or adjectival phrases of increasing difficulty, which participants have to produce as quickly as possible.	Reaction time	10 min	adapted from IDLaS-NL

21		Sentence generation	Participants see a written intransitive verb together with one argument, a transitive verb together with two arguments or a ditransitive verb with three arguments (nouns) on screen. They are instructed to produce a sentence using the verb and all of the displayed arguments in the order they appear from top to bottom within five seconds.	Accuracy	10 min	newly developed

22		Spontaneous speech	Participants speak freely for one minute about three topics provided (their dream holiday, a movie/book they watched/read, their activities during the last weekend).	Accuracy, reaction time	4 min	adapted from IDLaS-NL

23	Word comprehension	Rhyme judgment	Participants are presented with two non-words in succession and are instructed to judge as quickly as possible whether they rhyme.	Reaction time	5 min	adapted from IDLaS-NL

24		Auditory lexical decision	Participants judge the lexicality of an auditorily presented target word as quickly as possible.	Reaction time	7 min	adapted from IDLaS-NL

25		Semantic categorization	Participants judge as quickly as possible whether an auditorily presented target word belongs to a pre-specified semantic category.	Reaction time	5 min	adapted from IDLaS-NL

26	Sentence comprehension	Semantic match	Participants listen to a sentence and have to decide whether its content matches that of another sentence displayed on the screen.	Accuracy	8 min	newly developed

27		Comprehension questions	Participants listen to a sentence and have to answer a visually presented comprehension question about it by clicking on one of two possible answers displayed on screen.	Accuracy	8 min	newly developed

28.1		Gender cue identification	Participants indicate for 80 objects whether they take ‘der’ (masculine), ‘die’ (feminine) or ‘das’ (neuter) as their determiner. *It is advised to run this test preceding the test ‘Gender cue activation during sentence comprehension’*.	Accuracy	3 min	adapted from IDLaS-NL

28.2		Gender cue activation during sentence comprehension	Participants are presented with two objects (the same as in the Gender cue identification test) on the computer screen and a spoken sentence containing a target noun, which refers to one of the two objects. In half of the sentences, the target is predictable based on a determiner expressing the grammatical gender of the target noun. Participants indicate by button press which of the two objects is referred to in the sentence. On predictable trials, participants may respond before target noun onset. *It is advised to run this test following the test ‘Gender cue identification’*.	Reaction time	5 min	adapted from IDLaS-NL

29		Verb semantics activation during sentence comprehension	Participants are presented with two objects on the computer screen and a spoken sentence containing a target noun, which refers to one of the two objects. In half of the sentences, the target is predictable based on a verb expressing an action that can semantically only be carried out with the target noun. Participants indicate by button press which of the two objects is referred to in the sentence. On predictable trials, participants may respond before target noun onset.	Reaction time	5 min	adapted from IDLaS-NL

30	Extra test	Big Five Inventory 10	Participants rate ten statements about their personality.	Qualitative	5 min	Rammstedt et al. (2013)


All 35 tests of IDLaS-NL have been validated in a study with 748 Dutch-speaking participants and showed good construct validity (Hintz et al., 2025). All 30 tests in IDLaS-DE were piloted in five studies and validated in a sample of 181 participants. This sample completed the battery twice in order to assess test-retest reliability^1^ (Bethke et al., submitted). The results of the validation study are summarized in the appendix: Appendix A1 contains the descriptive statistics of all tests, including internal consistency and test-retest reliability values; Appendix A2 displays the correlations between the tests’ performance indicators. The psychometric measures overall show great response variability and internal consistency, as indicated by mainly good to excellent and only a few acceptable internal consistency values (range = 0.63–0.99). The test-retest reliability suggests good to excellent reliability for tests involving linguistic stimuli, with a few acceptable and little questionable cases (range = 0.57–0.92). The correlations between the tests’ performance indicators demonstrate that the different tests successfully assess individual variation in the eight psychological constructs they were designed to measure (see Appendix A2). The results thus indicate good construct validity of the IDLaS-DE test battery. These results and a detailed description of the tests are also available at the Max Planck Institute for Psycholinguistics Archive at: https://hdl.handle.net/1839/69596361-205d-4a51-8fbf-d817f032c781.

Both, IDLaS-NL and IDLaS-DE, are available as online platforms, on which researchers may create their own individual differences test batteries using any or all of the behavioral tests. The goal of the present article is to describe the German platform and provide guidance for its users.

## IDLaS-DE web platform – technical details

### Access

The IDLaS-DE test battery can be accessed via the project website www.mpi.nl/idlas-de. Researchers are directed to the web platform’s landing page, a PHP-based Graphical User Interface (GUI), which is accessible via https://ems13.mpi.nl/bq4_customizable_de/researchers_welcome.php.

### Frinex – framework for interactive experiment

The Frinex (**Fr**amework for **in**teractive **ex**periments) is a programming environment developed at the Max Planck Institute for Psycholinguistics (MPI) (Withers, 2016). All 30 tests were programmed in Frinex. Frinex deploys experiments as stand-alone tests with their own URL and their own experiment database. Experiments can be combined into one or multiple test sessions. All stimulus materials are downloaded before trial onset. The participant data are stored within the corresponding database. The stored data are tagged with study and researcher keys, with which researchers can retrieve the data from their study. All IDLaS-DE tests and data are stored on MPI servers. The servers hosting the experiment databases use GDPR-compliant SSL certificates for data transmission.

## IDLaS-DE web platform – a step-by-step guide

### Creating a study

**Figure 1:**
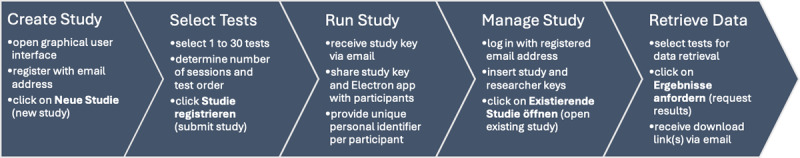
Step-by-step guide for creating, managing and running studies using the IDLaS-DE web platform (adapted from Hintz et al., 2024).

The flowchart in Figure 1 illustrates the five steps to create, run, and manage a study, including data retrieval, using the web platform (Figure 1). To create a customized IDLaS-DE study, researchers need to access the web platform at https://ems13.mpi.nl/bq4_customizable_de/researchers_welcome.php. Their email address serves as a user name. To register a study, researchers must accept the Terms of Use and provide the name of the city in which the Max Planck Institute for Psycholinguistics is located. This question works as a CAPTCHA to prevent bots from registering studies.

Researchers may create a new study by clicking on “Neue Studie” (New Study). This will guide them to the test selection page, where all 30 tests are listed. The tests are grouped by psychological construct. Next to the eight constructs mentioned in the Introduction, an additional category called “Extra test” includes a short version of the Big Five Inventory 10 (Rammstedt et al., 2013) that has not been developed by the IDLaS team, but is open access and can be used by interested researchers. For each test, there is a short task description (“i” icon) and an indication of the approximate duration of the test in minutes. Researchers can select tests they wish to include by ticking the box next to the test name. At the bottom of the page, they have to indicate how many sessions their study should be composed of (e.g., 1, 2 or more).

Clicking “Weiter” (Continue) takes researchers to the next page, where the test and session order can be determined. By clicking on the “- (löschen)” (remove) button and “+ (weiteren Test hinzufügen)” (add another test) button, tests can be removed or added to a session. All tests selected on the previous page will be listed in a drop-down menu next to each test number in a session. The test number refers to the position of the test within a session. The order of tests is determined by selecting a test from the drop-down menu for each position.

By clicking “Weiter” (Continue), the system performs a configuration check, evaluating whether all selected tests have been assigned to a session or whether tests occur more than once within a session. In such cases, the system will produce an error or warning message and researchers are prompted to make adjustments to their configuration. If the configuration check was successful, researchers finalize their study by clicking on “Studie registrieren” (submit study).

Upon registration of the study, a study key and a researcher key are generated and stored in our database together with the username (the email address provided). Researchers receive these keys and an overview of the study configuration via email. The researcher key serves as a password to log in to the platform with the username (e.g., to create a new study or retrieve experimental data). The study key serves as an identifier for the study and is needed (1) for participants to run the study, and (2) for researchers to retrieve the data associated with that study. Note that anyone with access to the registered email inbox can get access to both, the study and researcher keys.

### Modifying a study

To modify an existing study, researchers need to log in to the web platform (https://ems13.mpi.nl/bq4_customizable_de/researchers_welcome.php) with their username (i.e., the previously registered email address). Under the header “Existierende Studie” (existing study), the study key (Studienschlüssel) and researcher key (Researcher-Schlüssel) for the study researchers wish to modify need to be inserted. Clicking on “Existierende Studie öffnen” (open existing study) will provide researchers with three options: “Studie ändern” (modify study), “Ergebnisse anfordern” (request results), and “Studie löschen” (delete study). Clicking “Studie ändern” will open the same configuration page as for creating a new study. Selecting “Studie löschen” allows researchers to delete an existing study. Note that only the study configuration will be deleted, not the collected experimental data.

### Running a study

To run a study, researchers ought to consider some technical and practical details. These include the testing environment, hardware requirements, and relevant information to be shared with the participants.

#### Electron app for participant testing

All studies can either be run in a browser or in a dedicated testing environment designed for these purposes. This testing environment comprises an application that we provide to the researchers upon study registration. The application is built using Electron, a software framework for creating cross-platform desktop applications that run on multiple operating systems (OpenJS Foundation, 2023). More specifically, our Electron app is a Chromium-based browser that functions as a stand-alone application. It does not require any installation and can be run immediately after downloading. We recommend using Electron for following reasons: first, Electron reduces technical issues. Different participants use different browsers for running the study, which increases the risk of browser-related issues and variability. Since all tests were specifically designed to be run in Electron, running the study in the app minimizes the likelihood of technical issues.

Second, participants do not only use different browsers, but also operating systems, software and external devices. As timing precision for online experiments depends strongly on the soft- and hardware used (Anwyl-Irvine et al., 2021), using the Electron app reduces potential timing imprecision. A timing study conducted on six time-critical tests of IDLaS-NL showed similar precision as reported in Anwyl-Irvine et al. (2021): for auditorily presented stimuli, the playback was found to be delayed by 25 ms; keyboard responses were logged with a delay of approximately 100 ms. These delays were consistent across different operating systems (Monen et al., 2025).

Lastly, Electron takes up the whole computer screen, such that address line and tool bar are not visible. This way the study URL cannot be accessed or altered by participants and participants cannot switch between different tabs and get distracted.

Upon registration, researchers receive a READ ME file with practical information on how to run a study, including the download links for the Electron application. The Electron download links are available for Windows and Mac. In case the Electron app cannot be used, due to technical or other issues, we recommend using Google Chrome as a browser, since it runs on the same Chromium engine as Electron. To run a study via the browser, participants need to be directed to https://ems13.mpi.nl/bq4_customizable_de/index.html.

#### Hardware

All tests are designed to run on a laptop or computer. Mobile devices are not supported. Participants need a mouse(pad), keyboard, and an internal or external microphone, in case the study contains speech production tasks. It is advisable to use cable-bound headphones instead of the devices’ inbuilt speakers or wireless headphones. The latter typically come with a delay when playing back auditory stimuli, which affects the trial timing. To ensure that participants’ microphones are working properly and the quality of the sound recordings is sufficient, an audio- and microphone test is run by default before any session containing a speech production task. If the recording fails during the test or is noisy, changes need to be made to the microphone settings before the study begins. Sessions cannot be started until the microphone test has been passed.

#### Practical information for running a study

To run a study, researchers need to provide their participants with the following information: (1) the download link for the Electron app (Windows and Mac); (2) the study key associated with the study; and (3) a unique identifier for each participant. The identifier can be assigned by the researcher or generated by the participants themselves. Please instruct them to not use any personal information. The identifier needs to be at least three characters long. If the study consists of multiple sessions, researchers need to inform their participants about the time that needs to pass between the sessions.

Upon opening the Electron app, participants are asked to enter the study key and their personal identifier. The app retrieves the URL associated with the study. This URL contains all tests that are part of the study. The URL is not visible to the participants. Participants then select the session they wish to complete (previously completed sessions disappear from the list and cannot be selected). The tests that are part of the selected session will then be run in the designated order. After the last test of each session, participants are informed that the session has been completed and that they may now close the application. When reopening the app, the start screen is shown and participants can reenter the study key and their identifier to continue with the remaining sessions (if any). Note that closing the app in the middle of a study will mark the test that was running as completed. When reopening the app, participants may continue with the next test following the one that was aborted.

At the beginning of the first session of each study, a basic demographic questionnaire is run by default. Participants are asked to provide their age, gender, educational background, native language, handedness, and, if applicable, any medical issues. These data are anonymized and stored in a database separate from the experimental data. When requesting data for a test from the first (or only) session, the participants’ demographic data are provided as well. If researchers wish to collect additional demographic data beyond the basic information collected in this questionnaire, we advise to run a separate survey alongside the IDLaS-DE study.

### Data retrieval

To retrieve the experimental data, researchers need their researcher key and the study key associated with the study. Logging in to the web platform using their username and the corresponding keys, researchers can request the study data by clicking on “Ergebnisse anfordern” (request results). This is done for each test individually, or for all tests together. The results are sent via email. Result emails contain a download link (active for seven days) that leads to a zip file with the processed output. The email also contains a READ ME file describing the information listed in the different output files. For each test, there is a user response-file listing each participant’s response per item, and an irregularities-file containing information on potential issues, errors or irregularities during data collection or retrieval. For all accuracy-based tests, a scores-file provides a summary of the cumulative score per participant. For each speech production test, a zip folder is provided containing participants’ recordings per trial as wav-files. Where relevant, a stimulus-file is provided with information about the stimuli used in the respective test.

### Data usage

When using the web platform, our servers temporarily collect standard information from the user that ensures the usability of the webpage (e.g., IP address, date and time). Such data is stored separately from any personal data. The data will be deleted as soon as it is no longer required for the purpose for which it was collected (e.g., when terminating a session). Our servers used for data storage make use of GDPR-compliant SSL certificates for data transmission. Data collected in the demographic questionnaire as well as experimental data may be deleted upon request. Researchers and participants can request the deletion of data via email to idlas-de@mpi.nl. Our privacy policy is available on the welcome page of the web platform (https://ems13.mpi.nl/bq4_customizable_de/privacy.html).

## Further information

This article serves as a user guide for creating and running customized studies with IDLaS-DE. The GUI for IDLaS-DE was developed based on the GUI for IDLaS-NL, which means their functioning is identical. We therefore refer to Hintz et al. (2024) for further technical details and additional practical information, including recommendations for creating studies and testing participants, as well as for handling common technical issues (see also https://www.mpi.nl/idlas-de).

## Concluding remarks

IDLaS-DE is a test battery for assessing individual differences in linguistic experience, general cognitive abilities and linguistic processing skills, and was developed for German speaking adults. The web-based platform introduced in the present article offers a way of running individual differences studies in the lab or via the internet, allowing researchers to reach large and diverse participant samples. The battery is not intended for diagnostic purposes. IDLaS is already available in Dutch (accessible via https://www.mpi.nl/idlas-nl). An English version is currently under development. For updates on the English version please refer to https://www.mpi.nl/idlas-en.

## Data Accessibility Statement

The results of the validation study and a detailed description of the tests are available at the Max Planck Institute for Psycholinguistics Archive at: https://hdl.handle.net/1839/69596361-205d-4a51-8fbf-d817f032c781.

## Figures and Tables

**A2: Pearson’s correlation coefficients for all test scores.**
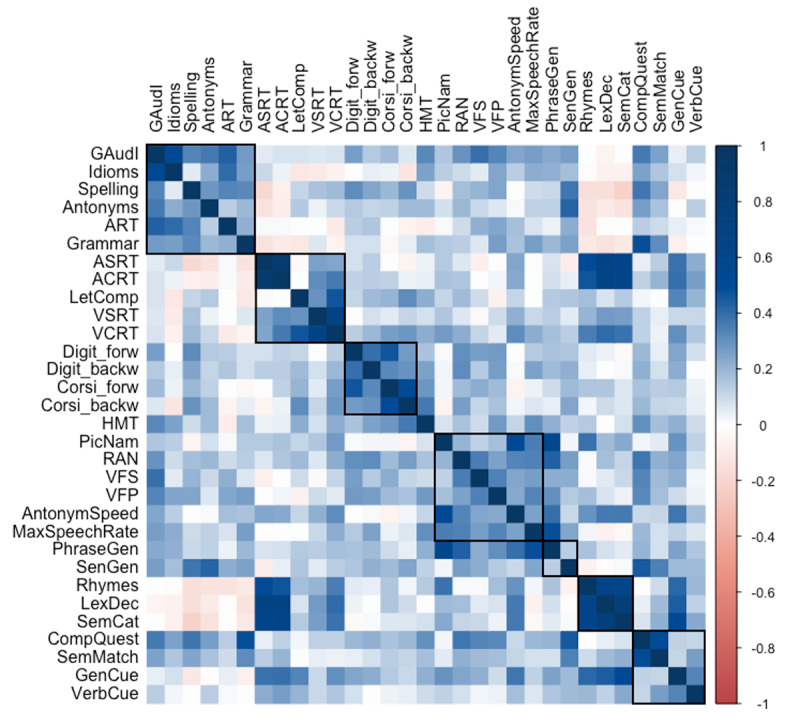
Coefficients included in the black squares belong to the same psychological construct: linguistic experience, processing speed, working memory, word production, sentence production, word comprehension, and sentence comprehension. Since there was only one test for nonverbal reasoning (Hagener Matrizentest, HMT), this test was not highlighted. Increasingly colder coloring (blue) represents strong positive correlations; increasingly warmer coloring (red) represents strong negative correlations.

## Footnotes

The second administration took place one month after completion of the first administration and was conducted on a subset of the IDLaS-DE test battery, excluding the eight tests measuring general cognitive skills. Since these eight tests were already validated in IDLaS-NL and do not contain any linguistic stimuli that differ between Dutch and German, there was no reason to assume that German speakers would perform differently than Dutch speakers.

## Appendix

**Table:** **A1: Descriptive statistics for all test scores**. N = number of participants after preprocessing, IC = internal consistency, TRR = test-retest reliability,^2^ Inv. log = inverse-coded (multiplied by -1) and log-transformed reaction times; raw reaction times are display in milliseconds.

TEST	N	MEAN (*SD*)	RANGE	SKEWNESS	KURTOSIS	IC	TRR

German Auditory and Image Vocabulary Test (GAudI)	174	0.74 (0.12)	0.38–0.95	–0.33	–0.33	0.87	0.92

Antonym Production (untimed)	180	0.83 (0.12)	0.20–1.00	–1.59	4.47	0.66	0.68

Idiom Recognition	174	0.78 (0.13)	0.32–1.00	–0.91	1.24	0.66	0.80

Spelling Test	181	0.52 (0.22)	0.00–1.00	–0.26	–0.49	0.82	0.82

German Author Recognition Test (ART)	179	0.26 (0.17)	0.00–0.86	0.77	0.35	0.93	–

Book Language Grammar Test	181	0.71 (0.09)	0.48–0.92	–0.11	–0.24	0.63	0.72

Auditory Simple Reaction Time test (A-SRT)	176	Inv. log: –2.55 (0.12)Raw: 372 (111)	Inv. log: –2.87––2.37Raw: 235–747	–0.8	–0.30	0.99	–

Auditory Choice Reaction Time test (A-CRT)	177	Inv. log: –2.72 (0.1)Raw: 546 (146)	Inv. log: –3.02––2.53Raw: 344–1075	–0.73	0.06	0.99	–

Letter Comparison	175	Inv. log: –3.03 (0.07)Raw: 1167 (192)	Inv. log: –3.25––2.83Raw: 698–1856	–0.22	0.34	0.96	–

Visual Simple Reaction Time test (V-SRT)	179	Inv. log: –2.45 (0.07)Raw: 286 (51)	Inv. log: –2.75––2.33Raw: 215–585	–1.13	2.03	0.95	–

Visual Choice Reaction Time test (V-CRT)	176	Inv. log: –2.66 (0.07)Raw: 469 (89)	Inv. log: –2.98––2.51Raw: 333–994	–1.23	2.72	0.95	–

Digit Span: forward	172	9.10 (2.21)	3–14	0.33	–0.28	0.77	–

Digit Span: backward	174	7.55. (2.35)	3–12	0.10	–0.69	0.76	–

Corsi Block Clicking test: forward	180	8.28 (2.10)	1–14	–0.09	0.83	0.72	–

Corsi Block Clicking test: backward	179	7.51 (2.18)	1–14	–0.01	0.58	0.75	–

Hagener Matrizentest	173	0.44 (0.20)	0.05–0.95	0.49	–0.42	0.79	–

Picture Naming	173	Inv. log: –3.00 (0.05)Raw: 1038 (133)	Inv. log: –3.13––2.86Raw: 725–1394	–0.04	–0.42	0.95	0.77

Rapid Automatized Naming	169	1.64 (0.24)	1.07–2.16	–0.07	–0.61	0.95	0.85

Verbal fluency: (semantic) categories	179	22.79 (4.72)	11.5–34.5	–0.01	–0.14	0.71	0.76

Verbal fluency: (phonemic) letters	179	13.13 (3.63)	3.5–21.5	0.16	–0.43	0.76	0.60

Antonym Production (timed)	162	Inv. log: –3.08 (0.07)Raw: 1269 (198)	Inv. log: –3.25––2.94Raw: 911–1835	–0.03	–0.5	0.93	0.76

Maximal Speech Rate	178	Inv. log: –3.65 (0.10)Raw: 4678 (1157)	Inv. log: –3.93––3.45Raw: 2820–8571	–0.52	–0.01	0.75	0.74

Phrase Generation	168	Inv. log: –3.24 (0.06)Raw: 1787 (254)	Inv. log: –3.42––3.12Raw: 1342–2748	–0.47	0.17	0.97	0.80

Sentence Generation	172	0.73 (0.18)	0.27–0.98	–0.74	–0.38	0.92	0.74

Rhyme Judgment	175	Inv. log: –2.98 (0.07)Raw: 992 (188)	Inv. log: –3.23––2.81Raw: 652–1743	–0.52	0.47	0.93	0.58

Auditory Lexical Decision	171	Inv. log: –2.95 (0.06)Raw: 917 (141)	Inv. log: –3.20––2.79Raw: 622–1652	0.76	1.35	0.98	0.68

Semantic Categorization	177	Inv. log: –2.99 (0.07)Raw: 1015 (167)	Inv. log: –3.25––2.81Raw: 655–1913	–0.42	0.86	0.97	0.58

Semantic Match	181	0.80 (0.08)	0.45–0.98	–0.99	2.28	0.64	0.64

Comprehension Questions	181	0.82 (0.1)	0.58–0.98	–0.52	–0.6	0.75	0.74

Gender cue activation during sentence comprehension	171	Inv. Raw: 418 (489)	–957–1335	–0.46	–0.33	0.93	0.79

Verb semantics activation during sentence comprehension	177	Inv. Raw: 533 (455)	–786–1263	–0.93	0.32	0.94	0.57
